# Crystalline Modification of Isotactic Polypropylene with a Rare Earth Nucleating Agent Based on Ultrasonic Vibration

**DOI:** 10.3390/polym11111777

**Published:** 2019-10-29

**Authors:** Dan Li, Yujun Xin, Yan Song, Ting Dong, Haoxi Ben, Renxia Yu, Guangting Han, Yuanming Zhang

**Affiliations:** 1 State Key Laboratory of Bio-Fibers and Eco-Textiles, Qingdao University, Qingdao 266071, China; dan950213@163.com (D.L.); xinyujun66@163.com (Y.X.); 18765915091@163.com (Y.S.); tingdong1988@gmail.com (T.D.); benhaoxi@163.com (H.B.); 19862802691@163.com (R.Y.); 2 College of Textile, Qingdao University, Qingdao 266071, China

**Keywords:** polypropylene, *β* nucleating agent, ultrasound, crystal behavior

## Abstract

In this paper, the crystalline modification of isotactic polypropylene (PP) with a rare earth *β* nucleating agent (WBG) with different ultrasound conditions was investigated by scanning electron microscopy (SEM), wide-angle X-ray diffraction (XRD), and differential scanning calorimetry (DSC). The relationship between the ultrasound conditions and the crystalline structure, as well as the mechanism for the behavior, were revealed. SEM showed that the dispersion of the nucleating agent in the PP matrix was better at shorter ultrasound distances. In addition, the higher the water cooling temperature, the better the nucleating agent was dispersed in the PP matrix. The results of XRD and DSC showed that the crystallinity and the relative content of the *β*-crystal were increased with nearer ultrasound distance, as well as increased in higher water cooling temperatures. In particular, under the same conditions, the crystallinity and the relative content of the *β*-crystal after ultrasonic treatment were much higher than those without ultrasound.

## 1. Introduction

Polypropylene (PP) is one of the most widely used plastics across the world because of its excellent performance and low price [1,2,3,4]. PP is a polymer with multiple crystalline phases [5,6], namely *α*-, *β*-, and *γ*-phases. It has been proven that the crystalline structure of PP has an important effect on its final properties. Among them, PP *α*- and *β*-phases are considered to have the highest application value. In general, *α*-phases show large grain size, compact structure, and poor anti-aging performance, while *β*-phases show small grain size, loose structure, and good anti-aging performance [7,8]. To date, many researchers have produced *β*-crystals by blending and melting [9,10,11] and grafting modifications [12,13,14]. Among these methods, addition of *β*-nucleating agent was considered to be promising because of the low cost, easier operation, and better results [15]. The rare earth *β* nucleating agent (WBG) is considered to be one of the most effective nucleating agents. The rare earth *β*-nucleating agent is an organic complex formed by combining one or more rare earth elements with one or more organic compounds [16,17]. The organic compound is usually selected from an organic heterocyclic compound, a fused ring compound, a fatty acid and a derivative thereof, an aromatic carboxylic acid and a derivative thereof, or other kinds of gems. The rare earth element is preferably a light rare earth element, such as cerium or lanthanum.

In addition, it has been found that ultrasound, as the carrier of energy transfer by mechanical vibration in elastic media, can induce the formation of the *β*-crystal form of polypropylene rare earth nucleating agent melt (WBG-PP) under certain conditions. High-intensity ultrasound can introduce a series of effects on the medium, such as a mechanical effect, thermal effect, cavitation effect, and acoustic flow effect [18,19,20,21]. Kang found that ultrasonic irradiation decreased the helical conformation order and changed crystalline structures [22]. Some researchers found that as the ultrasound intensity increased, the molecular weight of PP reduced and the distribution of its molecular weight became narrower; the orientation of PP molecules along the flow direction was reduced by applying the ultrasound vibration [18]. It is not difficult to understand that this was related to the effect of ultrasound on organic melts. However, to date, there has been little research on the effect of ultrasound on the crystallization behavior and mechanism of polypropylene. A more accurate understanding of the mechanism behind this is necessary to produce PP with high performance. Therefore, the purpose of this paper was to study the effect of ultrasound on the dispersion of the nucleating agent in the PP matrix, and the crystallinity behavior and crystallinity of WBG-PP.

In this paper, a kind of rare earth nucleating agent (WBG) was introduced into PP, and different ultrasound conditions were applied to increase the *β*-crystal content. The dispersion of the *β*-nucleating agent in the WBG-PP matrix and the crystallinity were characterized by scanning electron microscopy (SEM), X-ray diffraction (XRD), and differential scanning calorimetry (DSC).

## 2. Experimental Section

### 2.1. Materials

PP (trade name: F401) was purchased from Sinopec Yangzi Petrochemical Co., Ltd., Jiangsu, China, with a melt flow rate of 2.5 g/10min (230 °C, 21.6 N), a density of 0.91 g/cm^3^, and an equality of 96.5%. Rare earth *β*-nucleating agent WBG-II was kindly supplied by Yulinna Co., Guangdong, China.

### 2.2. Composites Preparation

**Figure 1:**
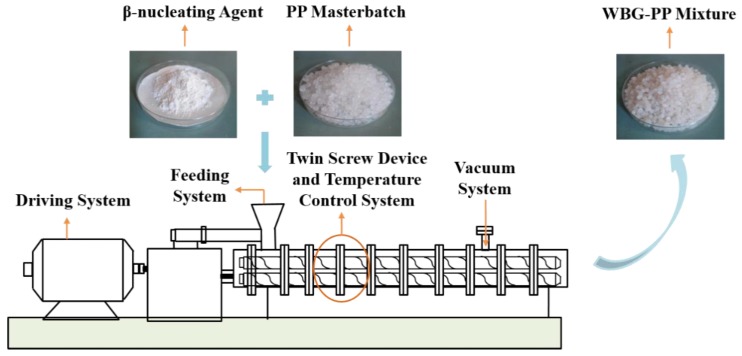
Process flow chart of preparing *β*-nucleating agent polypropylene (WBG-PP) using a twin-screw extruder.

Isotactic PP masterbatch was used as an original sample. Certain amounts of nucleating agent and PP were weighed separately using an electronic balance. Firstly, 1 g WBG nucleation and 100 g PP were mixed evenly in the discharging barrel of a twin-screw extruder equipped with a granulator. Then, the mixture was further prepared by mixing in the twin-screw extruder at a certain temperature. The process parameters are listed in Table 1, and the part of the process flow diagram of the twin-screw extruder is shown in Figure 1. The obtained blend particles were uniformly mixed with another 400 g PP and introduced into a twin-screw extruder to form extrusion granules. To ensure they were mixed evenly, the obtained granules were repeatedly introduced into a twin-screw machine (SHJ-20, Nanjing Jieente Electromechanical Co. Ltd., Nanjing, China) and cooled until granulated; thereby, a more uniform mixed sample particle was obtained.

### 2.3. Sample Prepared under Different Conditions

**Figure 2:**
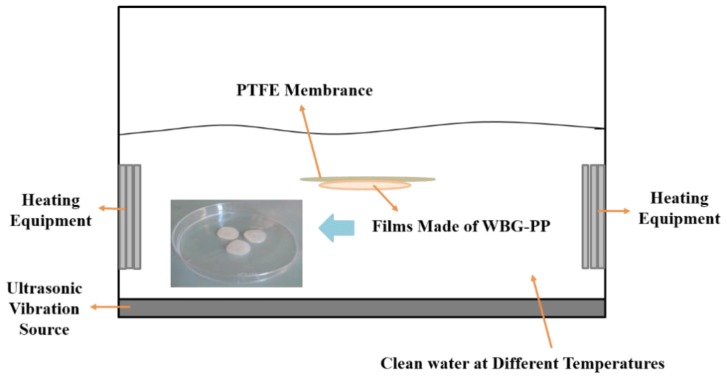
Device diagram of *β*-nucleating agent polypropylene (WBG-PP) thin film prepared by ultrasound.

The WBG-PP masterbatch was placed between two pieces of polytetrafluoroethylene (PTFE) film, and then placed on the digital thermostatic electric heating board. The temperature of the heating plate was set at 240 °C. After the masterbatch was completely melted, the melt was pressed into a 0.5 mm thin film. Then, the pressed melt was quickly put into the ultrasonic temperature-controlled water bath with one piece of PTFE film under special conditions. The blank samples were compared without ultrasound. The ultrasound device diagram is shown in Figure 2. The ultrasound was set at a certain frequency of 25 kHz and the power was 115 W. The distance between the modified polypropylene film and the vibration source was 1–7 cm and the water temperature was set to 30 to 70 °C.

**Figure 3:**
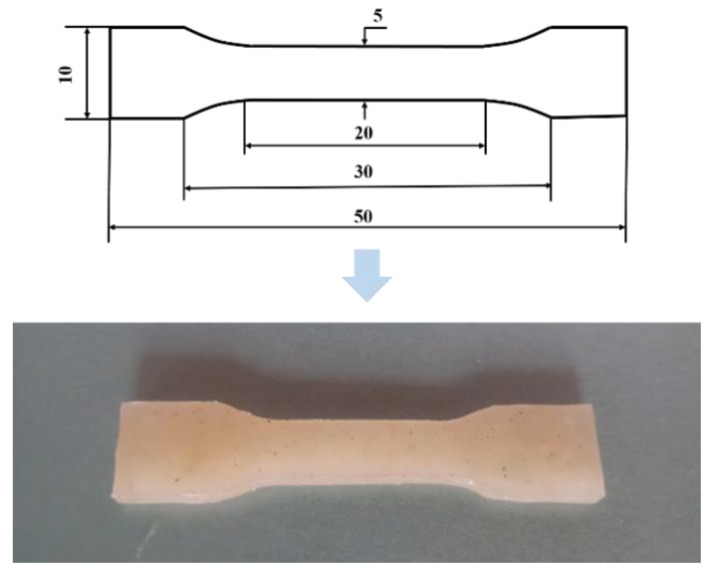
Shape and dimensions of a standard tensile spline.

The blended WBG-PP masterbatch was melted in a standard drawing dye, and once melted completely, the samples were quickly cooled in the ultrasonic equipment (XC-2000C, Jining Xinxin ultrasonic electronic equipment Co., Ltd., Jining, China). The prepared tensile spline is shown in Figure 3. The WBG-PP was tested with a tensile testing machine (BTKS5-10T150C, DongGuan Bell Experiment Equipment Co, Ltd., Dongguan, China) starting from room temperature and increasing at a rate of 50 mm/min.

### 2.4. Characterization

An X-ray diffractometer (Rigaku Smartlab (3KW), Rigaku Corporation, Tokyo, Japan) was used to irradiate Cuk*α* (*λ* = 0.1506 nm) at room temperature at an angle of 5–35° in the range of 2*θ*, and the scanning speed was 2°/min. According to Equations (1) and (2), the relative content of the *β*-crystal (*K_β_*) and the crystallinity (*w_c,x_*) of *β*-crystal can be calculated using the peak-splitting calculation method of computer diffraction curve fitting (Gaussian function). The equations used are [23,24,25]:

(1) $$K_{\beta }\;=\;\frac{I_{\beta }\left(300\right)}{I_{\alpha }\left(110\right)+I_{\alpha }\left(040\right)+I_{\alpha }\left(130\right)+I_{\beta }\left(300\right)}$$

(2) $$w_{c,x}\;=\;\frac{I_{c}}{I_{c}+k_{x}I_{x}}$$

In the formula, *I_β_*(300) is the integral intensity of the (300) diffraction of the *β*-crystal plane. The initial characteristic angles are 16.0. *I_α_* (110), *I_α_* (040), and *I_α_* (130) for the integral intensities of the (110), (040), and (130) diffractions, respectively, and the corresponding characteristic angles are 14.0, 16.8, and 18.6, respectively. *I_c_* is the integral intensity of crystal phase scattering, *I_x_* is the integral intensity of amorphous phase scattering, *k_x_* is the correction coefficient, and the value of *k_x_* is 1 [26].

The DSC results were employed to analyze the thermal properties of *β*-nucleated PP, including the melting behavior. Samples were taken at about 7 mg and indium was calibrated. The DSC tests were performed with TADSC250 differential scanning calorimetry (TravelCenters of America, New Castle, DE, USA) at a heating rate of 20 °C/min in a helium-protected environment and the temperature was raised to 240 °C. The heating curve was recorded. According to Equations (3) and (4), the relative content of the *β*-crystal form (*β_c_*) and crystallinity (*θ*) were calculated, respectively [27,28]:

(3) $$\theta \;=\;\frac{\Delta H_{f}}{\Delta H_{f}*}\;\times \;100\%$$

(4) $$\beta_{c}\;=\;\frac{X_{\beta }}{X_{\beta }+X_{\alpha }}\;=\;\frac{\frac{\Delta H_{\beta }}{\Delta H_{\beta {}^{\circ}}}}{\frac{\Delta H_{\beta }}{\Delta H_{\beta {}^{\circ}}}+\frac{\Delta H_{\alpha }}{\Delta H_{\alpha {}^{\circ}}}}$$

where $\Delta H_{f}$ is the melting heat of the sample and $\Delta H_{f}*$ is the melting heat of the polymer when its crystallinity reaches 100%; the value of $\Delta H_{f}*$ is 209 J/g [29]. Here, *X_α_* is the absolute crystallinity of *α*-crystal, and *X_β_* is the absolute crystallinity of the *β*–crystal. $\Delta H_{\beta \;}$ is the fusion heat caused by the *β*-crystal in the sample and $\Delta H_{\beta {}^{\circ}}$ is the standard fusion heat of *β*-crystal PP; the value of $\Delta H_{\beta {}^{\circ}}$ is 168.5 J/g [30]. Similarly, $\Delta H_{\alpha }$ is the fusion heat, which is caused by the *α*-crystal in the sample, and $\Delta H_{\alpha {}^{\circ}}$ is the standard fusion heat of *α*-crystal PP; the value of $\Delta H_{\alpha {}^{\circ}}$ is 177 J/g [30]. 

In order to study the dispersion of the *β*-nucleating agent in the PP matrix, scanning electron microscopy (SEM) (Jem-1200EX, Tokyo, Japan) was used to observe the micromorphology [31]. A BTKS5-10T150C tension machine (Dongguan Bell Experiment Equipment Co, Ltd., Dongguan, China) was used in the tensile tests.

## 3. Results and Discussion

### 3.1. The Effect of Different Ultrasound Condition on the Dispersion of β-Nucleating Agent in PP

In the PP processing, small particles of nucleating agent were uniformly dispersed in the PP melt, which provided a large number of heterogeneous nuclei for PP crystallization. PP molecules were adsorbed by nucleating agent through interfaces and the activation free energy required for nucleation and crystallization of PP by molecular chain self-motion agglomeration was reduced; thus, the crystallization rate was accelerated and the crystallization time was reduced [32].

**Figure 4:**
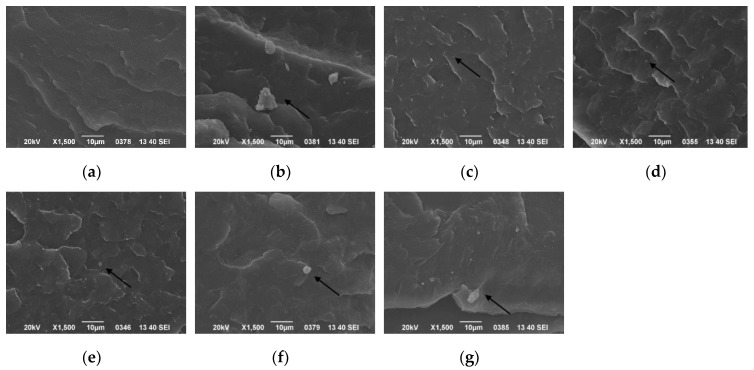
Effect of different ultrasound distances on dispersion of the nucleating agent in the PP matrix: (**a**) pure PP; (**b**) without ultrasound; (**c**) 1 cm; (**d**) 2 cm; (**e**) 3 cm; (**f**) 5 cm; (**g**) 7 cm.

The dispersion of *β*-nucleating agent in the PP matrix under different ultrasound conditions was monitored by SEM, as shown in Figure 4. It can be seen that pure PP had no obvious granular structure, but most nucleating agents were dispersed evenly in the PP matrix at short ultrasound distance. Most of the agents existed as primary particles, and the agglomeration phenomenon was greatly reduced. The nucleating agent particles adhered and stacked to form larger aggregates with the increase of ultrasound distance. Compared with WBG-PP without ultrasound, the dispersion of the nucleating agent was better with the application of ultrasound. The reason behind this was the synergistic effect of the thermal effect and acoustic flow effect caused by ultrasonic vibration. Under the action of ultrasonic stirring, the high-energy shear stress in the melt first tore the large aggregation into a small aggregation from the weak joint with smaller bond energy [33,34], which favored the dispersion of nucleating agent particles in the melt.

**Figure 5:**
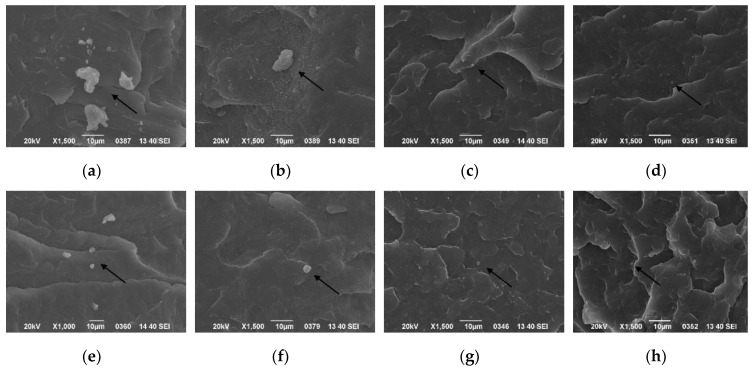
Dispersion of the nucleating agent under different cooling temperatures without ultrasound: (**a**) 30 °C, (**b**) 40 °C, (**c**) 50 °C, (**d**) 60 °C; and with ultrasound: (**e**) 30 °C, (**f**) 40 °C, (**g**) 50 °C, (**h**) 60 °C.

Figure 5 shows the dispersion of the nucleating agent in the PP matrix at different cooling temperatures, without ultrasound and with ultrasound application. It can be seen that the higher the cooling temperature, the better the nucleating agent was dispersed in the PP matrix, and the less agglomeration of the nucleating agent. At the same cooling temperatures, it also shows that the dispersion of the nucleating agent under ultrasound was better than that without ultrasound. This was attributed to the comprehensive effect of ultrasonic vibration, which favored the dispersion of nucleating agent particles.

### 3.2. The Effect of Ultrasound Distance on the Crystallinity of WBG-PP 

**Figure 6:**
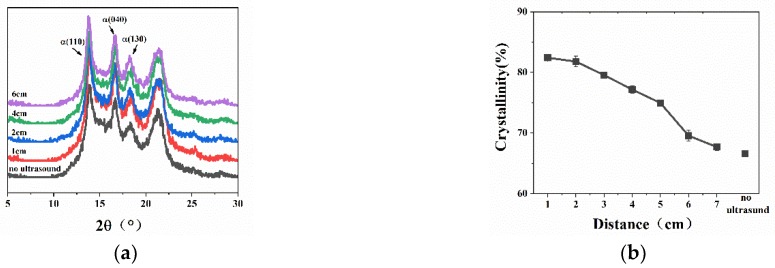
(**a**) Wide-angle X-ray diffraction pattern of pure PP prepared at different ultrasound distances. (**b**) Changes in the crystallinity (*w_c,x_*).

Figure 6 shows the wide-angle X-ray diffraction patterns of pure PP and the relationship with crystallinity at different ultrasound distances. From Figure 6a, it can be seen that when the water bath temperature was 70 °C, there were no *β* (300) crystal planes at different ultrasound distances. However, compared to the samples without ultrasound, the intensities of diffraction peaks of *α* (110), *α* (040), and *α* (130) crystal planes significantly increased in the samples with ultrasound. From Figure 6b, it can be seen that the crystallinity of pure PP with ultrasonic treatment was obviously higher than in the samples without ultrasound, and the closer the distance was, the higher the crystallinity. This phenomenon can be analyzed from a thermodynamic point of view. The energy generated by the ultrasonic vibration was absorbed by the melt when the short-distance ultrasound was applied, which slowed down the rate of cooling and prolonged the crystallization time. At the same time, in addition to ultrasonic vibration, the macromolecular chain in the melt had a certain orderliness in the amorphous phase region, which was conducive to the improvement of the crystallinity.

**Figure 7:**
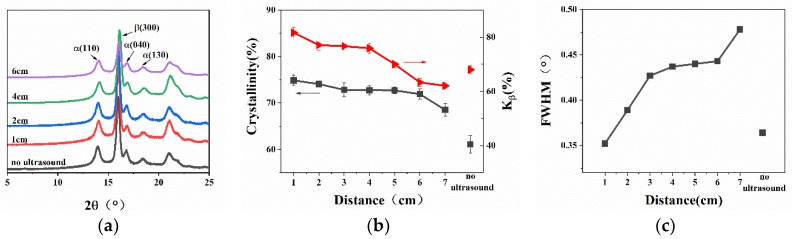
(**a**) Wide-angle X-ray diffraction pattern of WBG-PP prepared at different ultrasound distances. (**b**) Changes in the crystallinity (*w_c,x_*) and the relative content of *β*-crystals (*K_β_*). (**c**) Changes in the full width at half maximum (FWHM) of the *β* (300) crystal plane.

The XRD results of WBG-PP under the different ultrasound distance are shown in Figure 7. It can be seen in Figure 7a that the intensities of diffraction peaks of *α* (110), *α* (040), and *α* (130) were less affected by ultrasound, and the intensity of diffraction peaks of *β* (300) crystal planes increased at first and then weakened with further increase of distance. The results of XRD calculation showed that the crystallinity (*w_c,x_*) and the relative content of *β*-crystals (*K_β_*) increased significantly when ultrasound was applied compared with the samples without ultrasound. The values of *w_c,x_* and *K_β_* decreased predictably with the increase of ultrasound distance.

Apparently, the *w_c,x_* and *K_β_* values of WBG-PP increased after application of short-distance ultrasound. The temperature of the melt itself increased with the absorption of energy in the ultrasound process. This process slowed down the cooling rate of the melt and prolonged the crystallization time. In addition, when ultrasound was introduced, mechanical agitation due to the mechanical action turned the melt upside down in a short time. This significantly changed the temperature field of the pre-crystallization melt. The corresponding interface temperature gradient was increased, and the interfacial growth rate was reduced. Therefore, the *w_c,x_* and *K_β_* values of WBG-PP were increased. However, the values of *w_c,x_* and *K_β_* were decreased as the ultrasound distance increased. The ultrasound coefficient was affected by a variety of factors. Firstly, the attenuation of ultrasound waves in water is in accordance with the acoustic principle. According to the principle of sound attenuation, the intensity *P* of the ultrasound, whose initial intensity is *P*_0_, after the distance *x* can be expressed as [35]

*P* = *P*_0_exp(−*αx*) (5)

where *x* is the distance between the sound wave and the sound source and *α* is the attenuation coefficient.

With the increase of ultrasound distance, the absorption of ultrasonic energy by melt will lead to the decrease of sound intensity, and the attenuation coefficient was proportional to the ultrasound distance [35]. Secondly, with the increase of distance, the thermal effect transferred by ultrasound decreased, and the amount of heat absorbed by melt also decreased. Finally, the mechanical agitation of the ultrasound weakened as the distance increased. For the above reasons, the values of *w_c,x_* and *K_β_* for WBG-PP were decreased with the increase of ultrasound distance. In addition, it can be seen in Figure 7c that the full width at half maximum (FWHM) of the diffraction peak of the *β* (300) crystal plane increased with the increase of the ultrasound distance, which indicated that the nearer the distance, the better the crystallization was.

**Figure 8:**
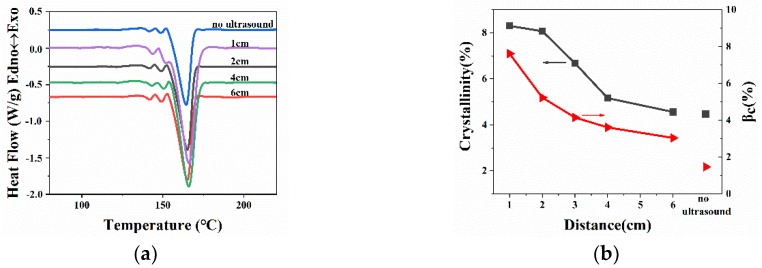
(**a**) The melting behaviors of WBG-PP at different ultrasound distances. (**b**) Effect of the ultrasound distance on the values of relative content of the *β*-crystal form (*β_c_*) and crystallinity (*θ*).

The DSC results for WBG-PP under nitrogen atmosphere are shown in Figure 8. Figure 8a shows the melting curves of WBG-PP at different ultrasound distances. It is known from the literature that the melting point of the *α*-crystal is about 176 °C. In these results, there were two peaks at 140 and 150 °C, which were due to the double peaks in the melting of the *β*-crystal [36]. The results of crystallinity calculation for WBG-PP are shown in Figure 8b. The *β*-crystal contents (*β_c_*) were calculated based on the melting curve and are listed in Table 2. It can be seen that the crystallinity (*θ*) and *β_c_* were decreased with the increase of ultrasound distance, and the value of *β_c_* and *θ* reached the maximum at the shortest distance.

### 3.3. The Effect of Ultrasound Temperature on the Crystallinity of WBG-PP

**Figure 9:**
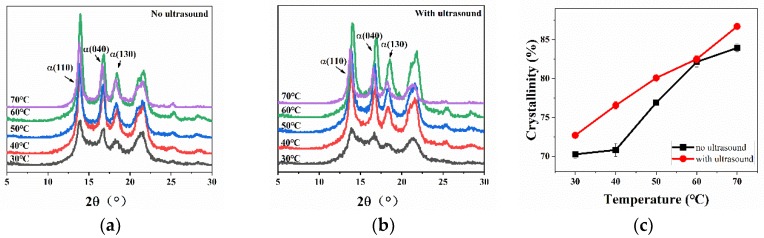
(**a**) X-ray diffraction (XRD) atlas of pure PP without ultrasound. (**b**) XRD atlas of pure PP with ultrasound. (**c**) The *w_c,x_* values of pure PP without ultrasound and with ultrasound.

Figure 9 shows wide-angle X-ray diffraction patterns of pure PP at different cooling temperatures and the changes of crystallinity. Comparing Figure 9a with Figure 9b, it can be seen that at the same cooling temperature, for the samples with ultrasound treatment applied, the intensities of the diffraction peaks of *α* (110), *α* (040), and *α* (130) crystal planes were obviously stronger than for samples without ultrasound. Figure 9c also shows that the crystallinity increased with the increase of cooling temperature, while the crystallinity of the samples treated with ultrasound was obviously higher than the samples without ultrasound. At 30 °C, the crystallinity was 2.444% higher than for the samples without ultrasound; at 50 °C, the crystallinity increased by 2.758%, and at 70 °C, the crystallinity increased by 3.174%. This shows that temperature and ultrasound had an effect on the crystallinity of pure PP. Ultrasound and high temperature were beneficial to the increase of crystallinity. At high cooling temperatures, the ultrasound was absorbed by the melt, and so the cooling rate was slow; thus, the crystallinity of the samples with ultrasound applied was improved.

**Figure 10:**
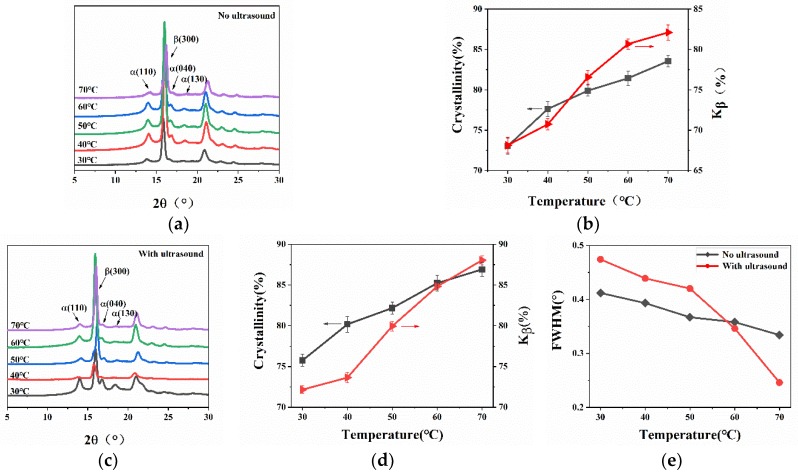
(**a**) XRD atlas of WBG-PP without ultrasound. (**b**) The *w_c,x_* and *K_β_* values of WBG-PP without ultrasound. (**c**) XRD atlas of WBG-PP with ultrasound. (**d**) The *w_c,x_* and *K_β_* values of WBG-PP with ultrasound. (**e**) Contrast of the FWHM of the diffraction peaks on the *β*-crystal plane with and without ultrasound.

The XRD curves were quite different for the WBG-PP cooled at the different water temperatures, as shown in Figure 10. In Figure 10a,c, it can be seen that the diffraction intensities of the *α* (110), *α* (040), *α* (130), and *β* (300) crystal planes were greatly affected by cooling temperature, and the increase of temperature was beneficial to the growth of the (300) crystal planes. The results of XRD calculation are shown in Figure 10b,d. With the increase of cooling temperature, the *w_c,x_* and *K_β_* values of WBG-PP increased. This is because the higher the cooling temperature, the longer the crystallization time was. Varga [37] and Lotz [38,39] showed that the optimum temperature for the growth of the *β*-crystal was 105–140 °C, in which the growth rate of the *β*-crystal was faster than that of the *α*-crystal. So, with the increase of cooling temperature, the residence time of the crystallization in the formation range of *β*-crystals was increased. In this time, *β*-crystals grew in a wider time range and the *β*-crystal content was increased. The value of *w_c,x_* increased with the increase of cooling temperature because the content of *α*- and *β*-crystals can be increased across the whole range of crystallization temperatures. It can be observed that in the samples with the application of ultrasound, the value of *w_c,x_* was significantly higher than for samples without ultrasound. The *β*-crystal content was 3.19% higher than for the samples without ultrasound at 30 °C. When the temperature was 60 °C, the *β*-crystal content of the samples with ultrasound was 4.14% higher than for the samples without ultrasound. This is because ultrasonic vibration and sound flow can produce circulation and turbulence in the melt, which causes the solidification process under conditions of intense motion, while without ultrasound can be considered as static [40]. In addition, the energy of the ultrasound was partially absorbed by the melt. These two effects tended to homogenize the temperature and concentration fields within the melt; thus, the dispersion of the nucleating agent in the PP matrix was more facilitated. Therefore, the crystallinity increased. From this point of view, the cooling temperature and ultrasound had a synergistic effect. Therefore, under the same cooling temperature environment, ultrasound was beneficial for improving the crystallinity. In addition, Figure 10e shows the change of the FWHM of the *β* (300) crystal plane with the increase of cooling temperature. From the figure, the FWHM of the *β*-crystal plane decreased obviously from 30 °C to 70 °C. This was attributed to the fact that the cooling rate decreased with the increase of cooling temperature, and the macromolecular chain had enough time to arrange into the crystal lattice to make the crystal structure complete.

**Figure 11:**
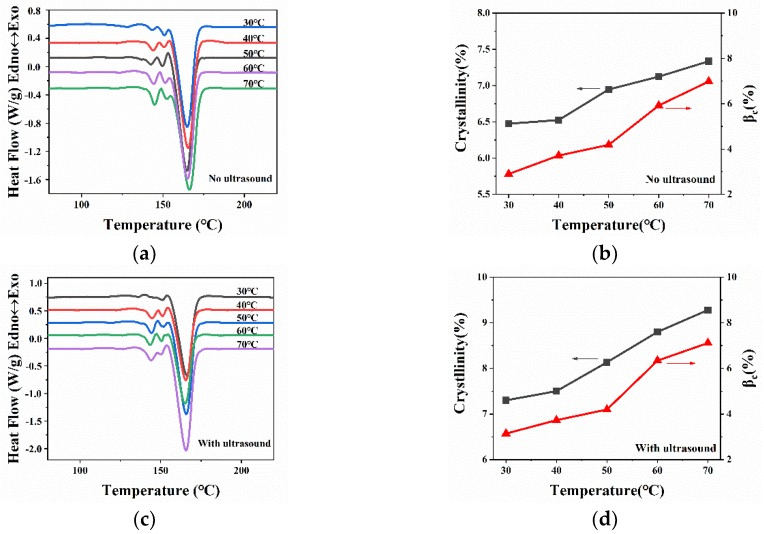
(**a**) The melting behaviors of WBG-PP under different cooling temperatures. (**b**) Effect of the cooling temperature on the values of *β_c_* and *θ*. (**c**) The melting behaviors of WBG-PP under different cooling temperatures with ultrasound. (**d**) Effect of the cooling temperature with ultrasound on the values of *β_c_* and *θ*.

Figure 11 shows the melting curves and crystallinity of WBG-PP at different ultrasound temperatures. It can be seen in Figure 11a,c that the area of the melting peak of the *β* phase increased gradually with the increase of cooling temperature, which led to the increase of *β*-crystal content. The values of *θ* and the *β*_c_ calculated based on melting curves are listed in Table 3. It can be seen that the values of *θ* and *β_c_* increased gradually with the increase of cooling temperature. This was consistent with the XRD results.

### 3.4. The Effect of Ultrasound Conditions on Mechanical Properties of WBG-PP

**Figure 12:**
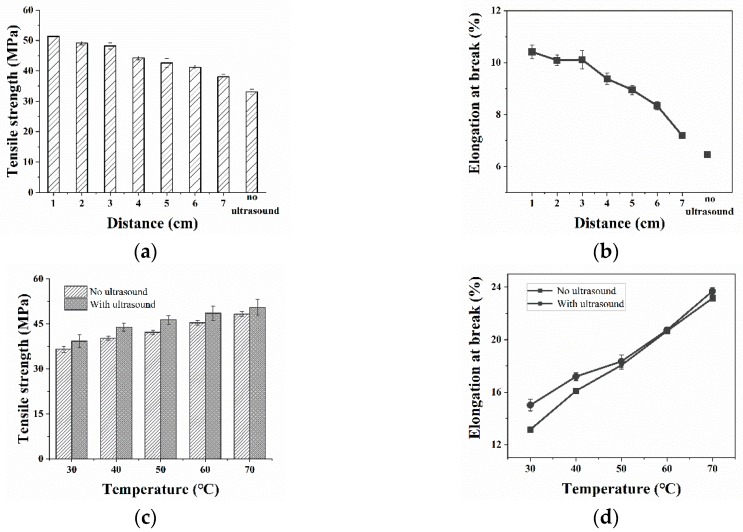
(**a**) The tensile strength of WBG-PP under different temperatures without ultrasound and with ultrasound. (**b**) The elongation at the break of WBG-PP under different temperatures without ultrasound and with ultrasound. (**c**) The tensile strength of WBG-PP at different ultrasound distances. (**d**) The elongation at the break of WBG-PP at different ultrasound distances.

The crystalline morphology of PP, especially the size, structure, crystallinity, and molecular orientation of spherulites, has significant influence on the tensile strength of PP parts. Due to the uneven grain distribution, pure PP tends to cause stress concentration and lower tensile strength under loading. Therefore, it is necessary to make the grain distribution uniform and reduce the stress concentration effect in order to improve the strength. The experimental results of the first two sections showed that the closer the ultrasonic distance and the higher the temperature, the better the dispensability of the *β*-nucleating agent in the PP matrix, and the slower the crystallization rate. Therefore, the grain size distribution was more uniform and finer. Thus, in this part, the cooling temperature was 70 °C, as the distance was variable; the ultrasonic distance was 1 cm, as the temperature was variable. The experimental results are shown in Figure 12.

Figure 12 shows the results of the tensile strength of WBG-PP under different ultrasound conditions. It can be seen in Figure 12a,b that the tensile strength of samples with ultrasound was higher than for the samples without ultrasound. The closer the ultrasound distance was, the greater the tensile strength. Because the content of the *β*-crystal decreased with the increase of ultrasound distance, the elongation at the break was decreased. Figure 12c,d shows that the tensile strength was increased with the increase of cooling temperature, and the tensile strength for samples with ultrasound applied was obviously higher than for samples without ultrasound. Combining the above conclusions, the higher that the cooling temperature was, the higher the content of *β*-crystal, and so the higher the elongation at the break. This shows that as the content of the *β*-crystal increased, the toughness of the material was also improved. Additionally, it shows that the increase of crystallinity resulted in the increase of the regular arrangement of components between molecular chains and the intermolecular force. This limits the movement of chain segments and increases the deformation resistance of the sheet.

## 4. Conclusions

This paper focuses on the crystallinity modification of WBG-PP under different ultrasound conditions, as well as the dispersion of *β*-nucleating agent in a PP matrix. The experimental results showed that short ultrasound distance was conducive to crystallinity and high tensile strength. When the ultrasound distance was 1 cm, we obtained products rich in *β*-nucleated PP, while the crystallinity reached the maximum. Additionally, the dispersion of *β*-nucleating agent in the PP matrix was the best. In addition, the crystallinity and tensile strength of WBG-PP were improved at higher cooling temperatures. The crystallinity after ultrasound was applied was obviously higher than without ultrasound, which indicates that there was a synergistic effect between ultrasound and cooling temperature. Besides, the higher that the cooling temperature was, the better the dispersion of *β*-nucleating agent in the PP matrix. This aggregation structure can affect the content of *β*-crystal and crystallinity morphology, owing to the efficiency of WBG.

## Figures and Tables

**Table 1:** Process parameters of *β*-nucleated polypropylene.

Temperature Zone	1	2	3	4
**Temperature Setting**	160	190	220	240
**Other Parameters**	The content of the *β*-nucleating agent	Twin-screw speed	Cooling temperature	Cooling time
	0.4%	60 r/min	20 °C	120 s

**Table 2:** The *β_c_* and *θ* values of WBG-PP at different ultrasound distances.

Sample	*X_α_* (%)	*X_β_* (%)	*β_c_* (%)	*θ* (%)
0	5.2050	0.0768	1.47	4.47
1	9.0887	0.7493	7.62	8.30
2	9.0600	0.5000	5.23	8.07
4	5.8800	0.2200	3.61	5.16
6	5.2250	0.1640	3.04	4.56

**Table 3:** The values of *β_c_* and *θ* of WBG-PP with different cooling temperatures without ultrasound and with ultrasound.

Sample	Without Ultrasound				With Ultrasound			
	*X_α_* (%)	*X_β_* (%)	*β_c_* (%)	*θ* (%)	*X_α_* (%)	*X_β_* (%)	*β_c_* (%)	*θ* (%)
30 °C	7.3762	0.2217	2.92	6.43	8.3626	0.2714	3.14	7.30
40 °C	7.4441	0.2848	3.68	6.57	8.5855	0.3326	3.73	7.50
50 °C	7.8146	0.3411	4.18	6.95	9.2790	0.4063	4.19	8.13
60 °C	7.8276	0.4891	5.88	7.17	9.9229	0.6716	6.34	8.80
70 °C	7.9046	0.5934	6.98	7.34	10.3914	0.7960	7.11	9.28

## References

[1] Galli P., Danesi S., Simonazzi T. (1984). Polypropylene based polymer blends: Fields of application and new trends. Polym. Eng. Sci..

[2] Shengjun J., Zongjian L. (1999). Status and development of polypropylene speciality for automobile in China. Chin. Synth. Resin Plast..

[3] Muñoz-Pascual S., Lopez-Gonzalez E., Saiz-Arroyo C., Rodriguez-Perez M.A. (2019). Effect of mold temperature on the impact behavior and morphology of injection molded foams based on polypropylene polyethylene-octene copolymer blends. Polymers.

[4] Gao M., Yang J., Zhao H., He H., Xie S. (2019). Preparation methods of polypropylene/nano-silica/styrene-ethylene-butylene-styrene composite and its effect on electrical properties. Polymers.

[5] Yuanming Z., Tingting S., Wei J., Han G. (2018). Crystalline modification of a rare earth nucleating agent for isotactic polypropylene based on its self-assembly. R. Soc. Open Sci..

[6] Housmans J.W., Gahleitner M., Peters G.W.M., Meijer H.E.H. (2009). Structure-property relations in molded, nucleated isotactic polypropylene. Polymer.

[7] Lotz B., Wittmann J.C., Lovinger A.J. (1996). Structure and morphology of poly(propylenes): A molecular analysis. Polymer.

[8] Yamamoto Y., Inoue Y., Onai T., Doshu C., Takahshi H., Uehara H. (2007). Deconvolution analyses of differential scanning calorimetry profiles of *β*-crystallized polypropylenes with synchronized X-ray measurements. Macromolecules.

[9] Wang K., Zhou C. (2010). The effects of melt vibration blending on the subsequent crystallization and melting behavior of polypropylene/ultra high molecular weight polyethylene. Polym. Eng. Sci..

[10] Lin Z., Guan Z., Xu B., Chen C., Guo G., Zhou J., Xian J., Gao L., Wang Y., Li M. (2013). Crystallization and melting behavior of polypropylene in *β*-PP/polyamide 6 blends containing PP-g-MA. J. Ind. Eng. Chem..

[11] Yang Z., Chen C., Liang D., Zhang Z., Mai K. (2009). Melting characteristic and *β*-crystal content of *β*-nucleated polypropylene/polyamide 6 alloys prepared using different compounding methods. Polym. Int..

[12] Wang J., Dong W., Zhu B., Dong Q., Zhang G. (2011). Advances in post-functionalzation of polypropylene by grafting modification. Chem. Ind. Eng. Prog..

[13] Han R., Nie M., Wang Q. (2015). Control over *β*-form hybrid shish-kebab crystals in polypropylene pipe via coupled effect of self-assembly *β* nucleating agent and rotation extrusion. J. Taiwan Inst. Chem. Eng..

[14] Wang D., Xie X.M., Jow J., Chen H.Y., Lai S.Y. (2008). Styrene-assisted melt free-radical grafting of pentaerythritol triacrylate onto polypropylene and its crystallization behavior. J. Appl. Polym. Sci..

[15] Ji H., Zhou X., Chen X., Zhao H., Wang Y., Zhu H., Shan X., Sha J., Ma Y., Xie L. (2019). Effects of solid-state stretching on microstructure evolution and physical properties of isotactic polypropylene sheets. Polymers.

[16] Feng J., Chen M., Huang Z., Guo Y., Hu H. (2002). Effects of mineral additives on the *β*-crystalline form of isotactic polypropylene. J. Appl. Polym. Sci..

[17] Varga J., Stoll K., Menyhárd A., Horváth Z. (2011). Crystallization of isotactic polypropylene in the presence of a *β*-nucleating agent based on a trisamide of trimesic acid. J. Appl. Polym. Sci..

[18] Chen J., Chen Y., Li H., Lai S., Jow J. (2010). Physical and chemical effects of ultrasound vibration on polymer melt in extrusion. Ultrason. Sonochem..

[19] Elgegren M., Kim S., Cordova D., Silva C., Noro J., Cavaco-Paulo A., Nakamatsu J. (2019). Ultrasound-assisted encapsulation of sacha inchi (plukenetia volubilis linneo.) oil in alginate-chitosan nanoparticles. Polymers.

[20] Eskin G.I., Pimenov Y.P., Makarov G.S. (1997). Effect of cavitation melt treatment on the structure refinement and property improvement in cast and deformed hypereutectic Al-Si alloys. Mater. Sci. Forum.

[21] Li Y.L., Ding H., Cao F.R. (2011). Effects of high density ultrasonic field coupling on the microstructures and properties of Al-Si alloy. Adv. Mater. Res..

[22] Kang J., Chen J., Cao Y., Li H. (2010). Effects of ultrasound on the conformation and crystallization behavior of isotactic polypropylene and *β*-isotactic polypropylene. Polymer.

[23] Turnerjones A., Cobbold A.J. (1968). The *β* crystalline form of isotactic polypropylene. J. Polym. Sci. Part B Polym. Lett..

[24] Jones A.T., Aizlewood J.M., Beckett D.R. (1964). Crystalline forms of isotactic polypropylene. Macromol. Chem. Phys..

[25] Hill R.J., Howard C.J. (1987). Quantitative phase analysis from neutron powder diffraction data using the Rietveld method. J. Appl. Crystallogr..

[26] Guien Z. (1989). X-ray Diffraction of Polymer.

[27] Li J., Cheung W. (1998). On the deformation mechanisms of *β*-polypropylene: 1. Effect of necking on *β*-phase PP crystals. Polymer.

[28] Shangguan Y., Song Y., Peng M., Li B., Zheng Q. (2005). Formation of *β*-crystal from nonisothermal crystallization of compression-molded isotactic polypropylene melt. Eur. Polym. J..

[29] Kargar-Kocsis J. (2012). Polypropylene: An AZ Reference.

[30] Li J.X., Cheung W.L., Jia D. (1999). A study on the heat of fusion of *β*-polypropylene. Polymer.

[31] Lai W.J., Cheng K.C. (2018). Crystallization and luminescence properties of polypropylene fiber containing rare earth aluminates and a sorbital derivative nucleating agent. Fibers Polym..

[32] Abdou J.P., Braggin G.A., Luo Y., Stevenson A.R., Chun D., Zhang S. (2015). Graphene-induced oriented interfacial microstructures in single fiber polymer composites. ACS Appl. Mater. Interfaces.

[33] Patel K.K., Kumar V., Purohit R., Gupta G.K., Modi O.P. (2017). Effect of ultrasonic stirring on changes in microstructure and mechanical properties of cast in-situ Al 5083 alloy composites containing 5wt.% and 10wt.% TiC particles. Mater. Today Proc..

[34] Dietemann M., Baillon F., Espitalier F., Calvet R., Accart P., Confetto S.D., Green-Hooper M. (2013). Evaluation of the physico-chemical properties of an amorphous magnesium silicate synthesized by an ultrasound-assisted precipitation. Chem. Eng. J..

[35] Heubner W., Eckardt J., Muller S. (2016). Ultrasonic sensor technology in hydraulic cylinders. ATZ Worldwide.

[36] Gao J.G., Yu M.S., Li Z.T. (2004). Nonisothermal crystallization kinetics and melting behavior of bimodal medium density polyethylene/low density polyethylene blends. Eur. Polym. J..

[37] Varga J. (1989). *β*-Modification of polypropylene and its two-component systems. J. Therm. Anal..

[38] Lotz B., Fillon B., Thierry A., Wittmann J.C. (1991). Low T_c_ growth transitions in isotactic polypropylene: *β* to *α* and *α* to smectic phases. Polym. Bull..

[39] Lotz B., Wittmann J.C. (1992). Isotactic polypropylene: Growth transitions and crystal polymorphism. Solidification Processes in Polymers.

[40] Jian X., Xu H., Meek T.T., Han Q. (2005). Effect of power ultrasound on solidification of aluminum A356 alloy. Mater. Lett..

